# Safety assessment of tranexamic acid: real-world adverse event analysis from the FAERS database

**DOI:** 10.3389/fphar.2024.1388138

**Published:** 2024-05-28

**Authors:** Ningsheng Tian, Yuxin Sun, Yingying Liu, Jie Jin, Shuai Chen, Huawei Han, Ying Zhang, Zhiwei Li

**Affiliations:** Department of Orthopaedics, The Second Affiliated Hospital of Nanjing University of Chinese Medicine, Nanjing, China

**Keywords:** tranexamic acid, FAERS, ADE, pharmacovigilance, drug discovery

## Abstract

**Background:**

In recent years, with the continuous expansion of the application scope of Tranexamic acid (TXA), its usage has surged. Despite numerous studies demonstrating its powerful efficacy, concerns regarding its adverse reactions persist, necessitating comprehensive safety assessment. This study analyzed real-world data from the U.S. Food and Drug Administration to investigate TXA-related adverse events, aiming to elucidate its safety and optimize patient treatment.

**Methods:**

The adverse drug event data concerning TXA from 2004 Q1 to 2023 Q3 were collected. Following data standardization, a variety of signal quantification techniques, including the reporting odds ratios, proportional reporting ratios, Bayesian confidence propagation neural network, and empirical Bayes geometric mean were used for analysis.

**Results:**

After analyzing 16,692,026 adverse event reports, a total of 1,574 cases of adverse events related to TXA were identified, spanning 23 system organ classes and 307 preferred terms. In addition to the common thrombosis-related Vascular disorders (*n* = 386) and Cardiac disorders (*n* = 377), adverse reactions in the Nervous system disorders category were also observed (*n* = 785), including Myoclonus (*n* = 70), Status epilepticus (*n* = 43), and Myoclonic epilepsy (*n* = 17). Furthermore, this study uncovered adverse effects such as Renal cortical necrosis, Hepatic cyst rupture, and Vascular stent stenosis, which were not previously mentioned in the instructions. Although these occurred infrequently, they exhibited high signal strength. Both Retinal artery occlusion and Vascular stent thrombosis disorder were frequent and exhibited high signal strength as well. It is worth noting that 78 cases of adverse reactions were caused by confusion between incorrect product administration.

**Conclusion:**

Our research suggests that TXA has some adverse reactions that are being overlooked. As a cornerstone medication in hemorrhage treatment, it’s crucial to monitor, identify, and address these adverse reactions effectively.

## 1 Introduction

### 1. Background

Since its development and release in the early 1960s, tranexamic acid (TXA), an indirect fibrinolytic inhibitor, has been utilized extensively. Initially, it was prescribed for heavy menstrual bleeding in females and individuals with hereditary bleeding disorders. Over time, its usage expanded to include elective surgery due to its effectiveness in reducing blood loss (Tengborn et al., 2015).

In recent years, there has been a steady uptick in the usage of TXA, with its applications continually broadening. TXA gained global recognition following the 2010 Clinical Randomization of an Antifibrinolytic in Significant Hemorrhage (CRASH-2) trial, with the result notably decreasing the risk of bleeding-related mortality by approximately one-sixth and overall mortality by about a 10th (Shakur et al., 2010). In 2011, the World Health Organization recommended TXA as an essential medicine for managing acute bleeding in patients with trauma, cardiopulmonary bypass, or *postpartum* hemorrhage (Sixty-third World Health Assembly, 2010). Subsequently, the 2013 European guidelines advocated for TXA’s use in prophylactic treatment during major surgeries to mitigate perioperative blood loss and allogeneic blood transfusion (Kozek-Langenecker et al., 2013). Additionally, the 2015 American Society of Anesthesiologists practice guidelines recommended considering TXA for surgical patients experiencing excessive bleeding (American Society of Anesthesiologists Task Force on Perioperative Blood Management, 2015). TXA has consistently demonstrated its significance in blood conservation and reducing perioperative blood loss across various medical domains, including trauma, cardiac, orthopedic, neurological, craniofacial, obstetrical, and gynecological surgeries (Ortmann et al., 2013).

Nevertheless, with the ongoing rise in utilization and expanding indications, numerous inquiries persist regarding the additional clinical effects of TXA. These include its potential anti-inflammatory response during cardiopulmonary bypass, the risk of thromboembolic events, adverse neurological effects such as seizures, as well as its incidence and mortality rates (Ortmann et al., 2013). Hence, there is an urgent necessity to thoroughly assess the safety profile of TXA across various medical disciplines. In this data analysis, we utilize real-world pharmacovigilance data, predominantly from the U.S. Food and Drug Administration (FDA) Adverse Event Reporting System (FAERS), to scrutinize adverse events (AEs) linked with TXA administration. Through meticulous examination of these reports, our objective is to illuminate the safety landscape of TXA, pinpoint potential risk factors, and offer valuable insights to clinicians for optimizing patient care strategies.

## 2 Materials and methods

### 2.1 Data source

Given TXA’s market release date, this study retrieved the American Standard Code for Information Interchange (ASCII) report files from the FAERS database spanning from the first quarter of 2004 to the third quarter of 2023. The data underwent processing using R_4.3.2 software.

### 2.2 Data extraction and analysis

**FIGURE 1:**
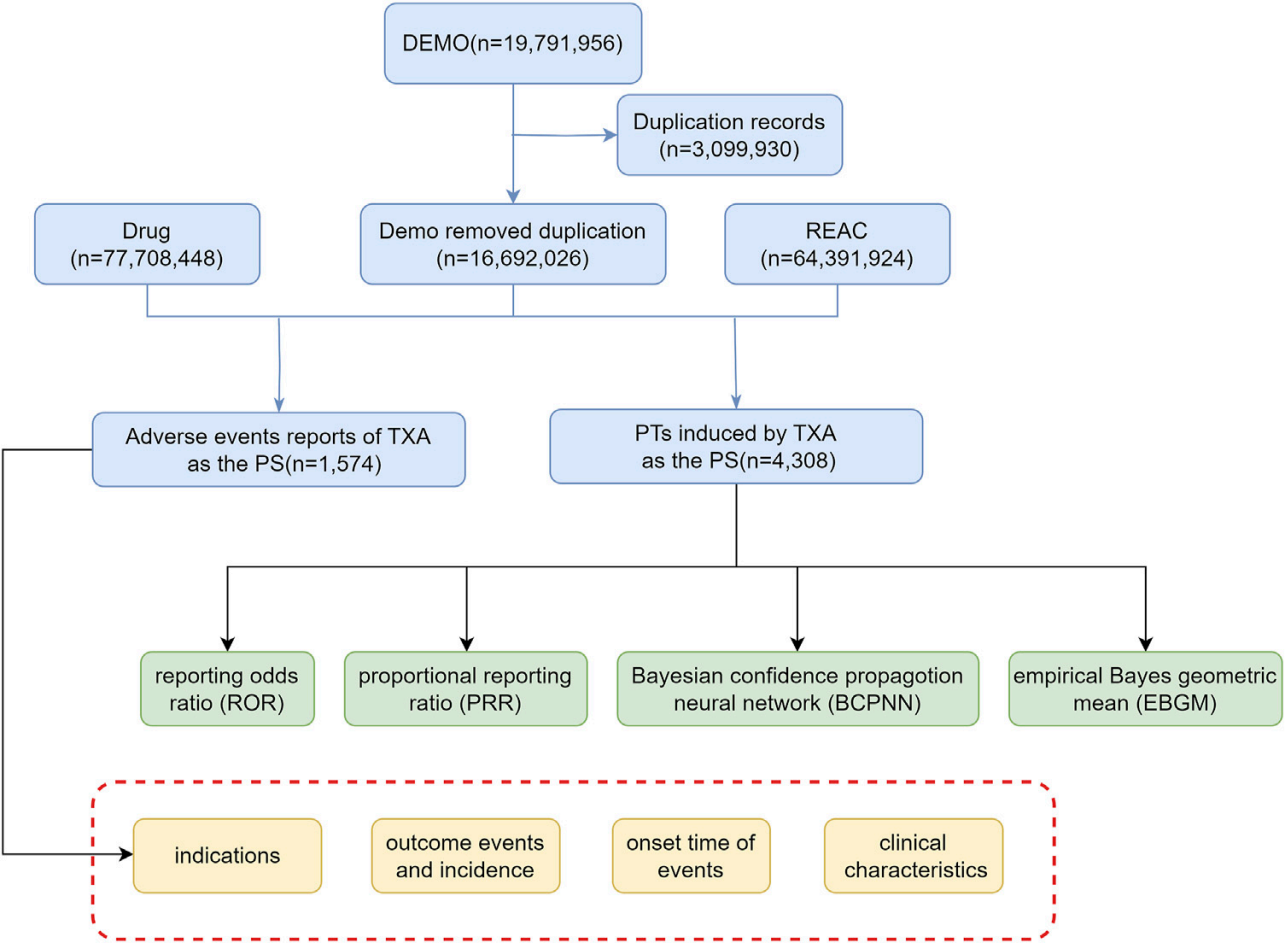
The flow diagram of selecting TXA-related AEs from FAERS database.

To ensure data integrity, duplicate reports were removed. For reports sharing the same CASE number, the latest FDA_DT is chosen. In cases where both the CASE number and FDA_DT are identical, the report with the higher ISR number is selected. Screening is conducted based on the drug name: tranexamic acid, with the suspicion level of reports limited to “Primary Suspect (PS)”. Finally, we obtain adverse event reports (AERs) of TXA and preferred terms (PTs) induced by TXA (Figure 1). The relationships among datasets were established using the primaryid field. Standardization of drug names was achieved through the Medex_UIMA_1.8.3 system.

This study simultaneously employed the reporting odds ratios (ROR) (Rothman et al., 2004), proportional reporting ratios (PRR) (Evans et al., 2001), Bayesian confidence propagation neural network (BCPNN) (Bate et al., 1998), and empirical Bayes geometric mean (EBGM) (Dumouchel, 1999) techniques from the disproportionality analysis to detect ADEs. Through their joint application and cross-validation to reduce potential false positives, thresholds and variances were adjusted to enhance the detection of potentially rare adverse reactions. All algorithms were based on 2 × 2 contingency tables, as shown in Supplementary Table S1. Specific formulas and thresholds are outlined in Supplementary Table S2. Statistical analysis was conducted using R_4.3.2 software. Higher values indicate stronger signal intensity, suggesting a stronger association between the target drug and AEs.

### 2.3 Signal filtering and categorization

PTs with reporting counts of ≥3 were selected. The Medical Dictionary for Regulatory Activities (MedDRA) PTs and System Organ Classes (SOCs) were utilized to encode, classify, and localize signals, facilitating the analysis of specific SOCs related to AE signals.

## 3 Results

### 3.1 Basic characteristics of TXA-related ADEs

**FIGURE 2:**
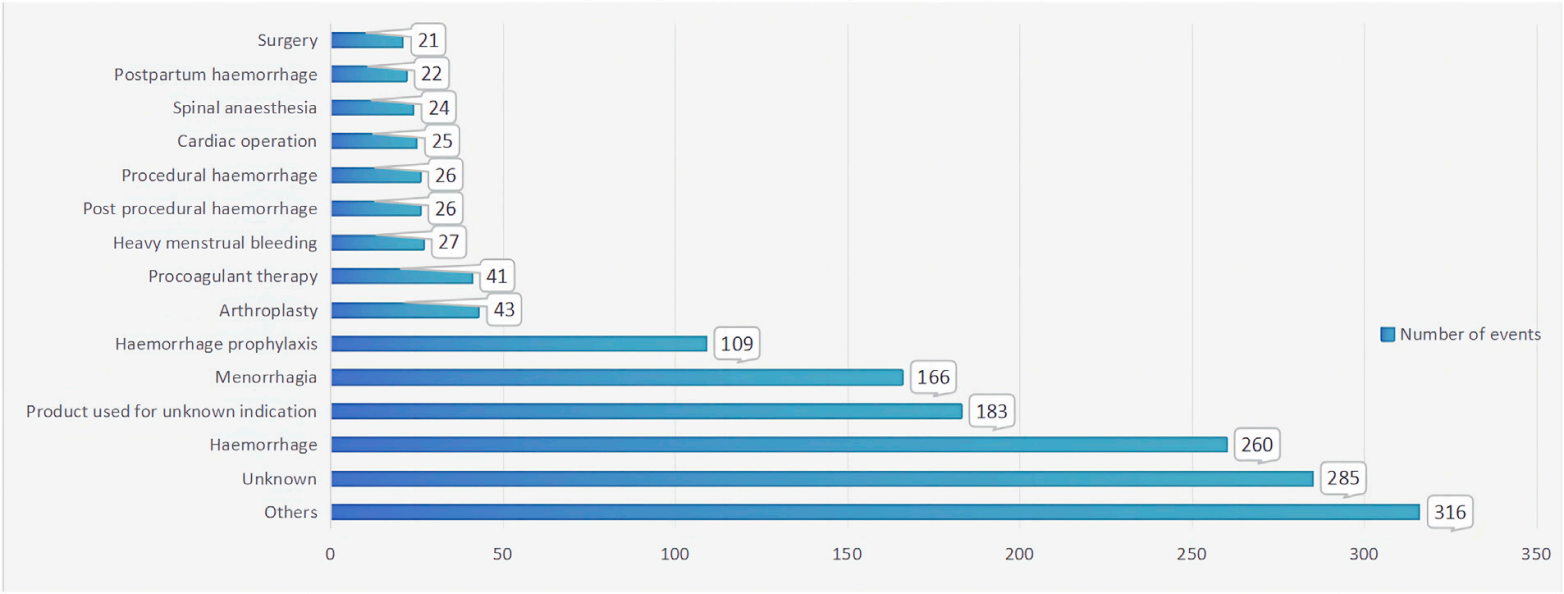
The indications on ADEs and PTs related to TXA.

From the first quarter of 2004 to the third quarter of 2023, this study obtained a total of 16,692,026 AERs from the FAERS database. Among these reports, TXA was identified as the primary suspected drug for ADEs in 1,574 cases. Interestingly, fluctuations in the annual distribution of these reports were observed, with a notable surge in recent years, peaking in 2019 with 283 reports, representing 17.98% of the total. Gender analysis revealed a significant skew towards female patients, constituting 52.99% of the reports, compared to 29.73% for male patients. Across different age groups, the distribution of reports was relatively uniform, without any discernible variations. Notably, the majority of reports were submitted by healthcare professionals, predominantly physicians (36.21%) and pharmacists (27.89%), with consumer reports making up 9.78% of the total submissions. Geographically, the United States contributed the highest proportion of reports at 31.89%, followed by a diverse international representation. Various administration routes were documented, with intravenous (26.56%) and oral (16.26%) routes being the most prevalent. Notably, serious outcomes associated with ADEs, such as hospitalization (24.29%), life-threatening conditions (11.09%), and death (8.01%), underscore the importance of vigilant pharmacovigilance. Additionally, the majority of ADEs occurred within 7 days of medication usage, as revealed by our investigation. TXA, known for its diverse indications, was primarily associated with hemorrhage (16.52%) and menorrhagia (10.54%). Details can be found in Table 1 and Figure 2.

**TABLE 1:** Basic information on ADEs related to TXA from the FAERS database.

Factors	Number of events (%)
Year	
2004	15 (0.95)
2005	10 (0.64)
2006	3 (0.19)
2007	5 (0.32)
2008	16 (1.02)
2009	13 (0.83)
2010	60 (3.81)
2011	38 (2.41)
2012	65 (4.13)
2013	60 (3.81)
2014	46 (2.92)
2015	80 (5.08)
2016	82 (5.21)
2017	100 (6.35)
2018	112 (7.12)
2019	283 (17.98)
2020	199 (12.64)
2021	160 (10.17)
2022	122 (7.75)
2023	105 (6.67)
Gender	
Female	834 (52.99)
Male	468 (29.73)
Unkown	272 (17.28)
Age	
<18	101 (6.42)
18–44	310 (19.70)
45–64	347 (22.05)
65–74	190 (12.07)
≥75	228 (14.49)
Unknow	398 (25.29)
Reporter	
Physician	570 (36.21)
Pharmacist	439 (27.89)
Other health-professional	326 (20.71)
Consumer	154 (9.78)
Unkown	83 (5.27)
Lawyer	2 (0.13)
Reported countries	
United States	502 (31.89)
Others	444 (28.21)
United Kingdom	303 (19.25)
Germany	97 (6.16)
Netherlands	59 (3.75)
Japan	54 (3.43)
France	33 (2.10)
Canada	28 (1.78)
India	28 (1.78)
Spain	26 (1.65)
route	
Others	818 (51.97)
Intravenous	418 (26.56)
Oral	256 (16.26)
Intrathecal	82 (5.21)
Serious outcomes	
Other serious	1058 (53.33)
Hospitalization	482 (24.29)
Life threatening	220 (11.09)
Death	159 (8.01)
Disability	55 (2.77)
Adverse event occurrence time - medication date (days	
<7	285 (18.11)
7–28	35 (2.22)
≥28	40 (2.54)
Unknow	1214 (77.13)
Indications	
Others	316 (20.08)
Unknown	285 (18.11)
Haemorrhage	260 (16.52)
Product used for unknown indication	183 (11.63)
Menorrhagia	166 (10.54)
Haemorrhage prophylaxis	109 (6.93)
Arthroplasty	43 (2.73)
Procoagulant therapy	41 (2.60)
Heavy menstrual bleeding	27 (1.72)
Post procedural haemorrhage	26 (1.65)
Procedural haemorrhage	26 (1.65)
Cardiac operation	25 (1.59)
Spinal anaesthesia	24 (1.52)
*Postartum* haemorrhage	22 (1.40)
Surgery	21 (1.33)

### 3.2 TXA signal mining

This study found that AEs related to TXA involved 23 System Organ Classes (SOCs). The three most common systems were Nervous system disorders (*n* = 785, ROR 2.41, PRR 2.14, IC 1.1, EBGM 2.14), Injury, poisoning and procedural complications (*n* = 529, ROR 1.42, PRR 1.36, IC 0.45, EBGM 1.36), and General disorders and administration site conditions (n = 437, ROR 0.52, PRR 0.57, IC −0.8, EBGM 0.57). Furthermore, Vascular disorders (*n* = 386, ROR 4.49, PRR 4.16, IC 2.06, EBGM 4.16) and Cardiac disorders (*n* = 377, ROR 3.65, PRR 3.4, IC 1.77, EBGM 3.4) exhibited the strongest signal intensities. Details can be found in Table 2.

**TABLE 2:** The signal strength of ADEs of TXA at the SOC level in FAERS database.

System organ class	Case reports	ROR (95% CI)	PRR (95% CI)	χ^2^	IC(IC025)	EBGM (EBGM05)
Vascular disorders	386	4.49 (4.04, 4.98)	4.16 (3.77, 4.59)	947.15	2.06 (1.91)	4.16 (3.81)
Cardiac disorders	377	3.65 (3.28, 4.05)	3.4 (3.08, 3.75)	657.44	1.77 (1.61)	3.4 (3.11)
Nervous system disorders	785	2.41 (2.23, 2.6)	2.14 (2.02, 2.27)	521.36	1.1 (0.99)	2.14 (2)
Renal and urinary disorders	131	1.73 (1.45, 2.06)	1.71 (1.43, 2.04)	38.91	0.77 (0.52)	1.7 (1.47)
Immune system disorders	76	1.59 (1.27, 1.99)	1.58 (1.27, 1.96)	16.3	0.66 (0.33)	1.58 (1.31)
Respiratory, thoracic and mediastinal disorders	310	1.58 (1.4, 1.77)	1.53 (1.39, 1.69)	60.16	0.61 (0.45)	1.53 (1.39)
Reproductive system and breast disorders	54	1.51 (1.16, 1.98)	1.51 (1.15, 1.99)	9.24	0.59 (0.21)	1.51 (1.2)
Injury, poisoning and procedural complications	529	1.42 (1.29, 1.55)	1.36 (1.26, 1.47)	56.91	0.45 (0.32)	1.36 (1.26)
Blood and lymphatic system disorders	92	1.29 (1.05, 1.58)	1.28 (1.05, 1.56)	5.76	0.36 (0.06)	1.28 (1.08)
Eye disorders	89	1.04 (0.84, 1.28)	1.03 (0.85, 1.25)	0.11	0.05 (−0.25)	1.03 (0.87)
Pregnancy, puerperium and perinatal conditions	16	0.83 (0.5, 1.35)	0.83 (0.51, 1.35)	0.59	−0.28 (−0.96)	0.83 (0.55)
Investigations	202	0.77 (0.66, 0.88)	0.78 (0.68, 0.89)	13.84	−0.36 (−0.57)	0.78 (0.69)
Hepatobiliary disorders	27	0.71 (0.49, 1.04)	0.72 (0.5, 1.04)	3.08	−0.48 (−1.02)	0.72 (0.52)
Skin and subcutaneous tissue disorders	141	0.59 (0.5, 0.7)	0.6 (0.51, 0.7)	39.23	−0.73 (−0.97)	0.6 (0.52)
Musculoskeletal and connective tissue disorders	130	0.57 (0.48, 0.68)	0.59 (0.49, 0.7)	39.95	−0.77 (−1.02)	0.59 (0.51)
Congenital, familial and genetic disorders	7	0.54 (0.26, 1.13)	0.54 (0.26, 1.14)	2.78	−0.89 (−1.89)	0.54 (0.29)
General disorders and administration site conditions	437	0.52 (0.47, 0.58)	0.57 (0.52, 0.63)	169.86	−0.8 (−0.94)	0.57 (0.53)
Ear and labyrinth disorders	9	0.49 (0.26, 0.94)	0.49 (0.26, 0.94)	4.75	−1.02 (−1.92)	0.49 (0.28)
Metabolism and nutrition disorders	42	0.46 (0.34, 0.62)	0.46 (0.34, 0.62)	26.63	−1.11 (−1.54)	0.46 (0.36)
Gastrointestinal disorders	147	0.38 (0.32, 0.45)	0.4 (0.34, 0.47)	145.09	−1.32 (−1.56)	0.4 (0.35)
Psychiatric disorders	62	0.24 (0.19, 0.31)	0.25 (0.19, 0.32)	147.58	−1.99 (−2.35)	0.25 (0.2)
Infections and infestations	35	0.15 (0.11, 0.21)	0.16 (0.11, 0.22)	163.77	−2.65 (−3.12)	0.16 (0.12)
Neoplasms benign, malignant and unspecified (incl cysts and polyps)	15	0.12 (0.07, 0.2)	0.13 (0.08, 0.22)	93.54	−2.99 (−3.7)	0.13 (0.08)

A total of 307 PTs, with the top 30 PTs ranked by the ROR algorithm displayed in Table 3. The results indicated that Ureteric perforation (*n* = 5, ROR 1773.55, PRR 1771.38, IC 10.57, EBGM 1518.47), Myoclonic epilepsy (*n* = 17, ROR 174.12, PRR 172.4, IC 7.41, EBGM 170.63), and Superficial vein thrombosis (*n* = 4, ROR 158.79, PRR 158.63, IC 7.29, EBGM 156.31) were high signal focal points. The most common AEs were Myoclonus (*n* = 70, ROR 82.38, PRR 80.99, IC 6.33, EBGM 80.39) and Status epilepticus (*n* = 43, ROR 53.68, PRR 53.12, IC 5.72, EBGM 52.86). In addition to adverse effects mentioned in the product label, this study also discovered rare but high signal intensity adverse events such as Renal cortical necrosis, Hepatic cyst ruptured, and Vascular stent stenosis. Both Retinal artery occlusion and Vascular stent thrombosis disorder were observed with high frequencies and signal intensities. Notably, there were 78 instances of injury, poisoning, and procedural complications attributed to Tranexamic acid, stemming from incidents of wrong product administration, confusion regarding product appearance, and ambiguity in product packaging.

**TABLE 3:** The top 30 signal strength of AEs of TXA ranked by ROR at the PTs level in FAERS database.

SOC	PTs	Case reports	ROR (95% CI)	PRR (95% CI)	χ2	IC (IC025)	EBGM (EBGM05)
Nervous system disorders	Myoclonic epilepsy	17	174.12 (107.72, 281.45)	173.4 (108.33, 277.55)	2867.17	7.41 (6.74)	170.63 (114.17)
Nervous system disorders	Cerebral artery thrombosis	4	100.36 (37.48, 268.78)	100.27 (37.63, 267.17)	389.44	6.63 (5.36)	99.34 (43.57)
Nervous system disorders	Cauda equina syndrome	5	96.39 (39.94, 232.62)	96.27 (39.85, 232.56)	467.18	6.58 (5.41)	95.42 (45.65)
Nervous system disorders	Myoclonus	70	82.38 (64.99, 104.43)	80.99 (64.02, 102.47)	5489.56	6.33 (5.99)	80.39 (65.92)
Nervous system disorders	Orthostatic intolerance	4	74.92 (28.01, 200.4)	74.85 (28.09, 199.43)	289.41	6.22 (4.95)	74.33 (32.63)
Nervous system disorders	Cerebral venous sinus thrombosis	3	71.38 (22.93, 222.26)	71.33 (22.89, 222.32)	206.65	6.15 (4.73)	70.86 (27.4)
Nervous system disorders	Ischaemic cerebral infarction	5	65.36 (27.12, 157.55)	65.28 (27.02, 157.7)	314.57	6.02 (4.86)	64.89 (31.08)
Nervous system disorders	Status epilepticus	43	53.68 (39.72, 72.54)	53.12 (39.59, 71.28)	2188.6	5.72 (5.29)	52.86 (41.09)
Vascular disorders	Superficial vein thrombosis	4	158.79 (59.13, 426.37)	158.63 (59.54, 422.66)	617.34	7.29 (6.01)	156.31 (68.4)
Vascular disorders	Vasodilatation	16	90.04 (54.99, 147.41)	89.69 (54.95, 146.4)	1391.54	6.47 (5.78)	88.95 (58.88)
Vascular disorders	Peripheral artery thrombosis	8	80.6 (40.17, 161.7)	80.44 (40.51, 159.73)	622.93	6.32 (5.37)	79.84 (44.59)
Vascular disorders	Arterial thrombosis	8	55.25 (27.56, 110.75)	55.14 (27.77, 109.49)	423.09	5.78 (4.83)	54.86 (30.66)
Injury, poisoning and procedural complications	Wrong product administered	55	135.51 (103.68, 177.11)	133.7 (103.63, 172.5)	7154.9	7.05 (6.66)	132.06 (105.55)
Injury, poisoning and procedural complications	Product appearance confusion	8	121.53 (60.5, 244.14)	121.29 (59.89, 245.62)	943.66	6.91 (5.96)	119.94 (66.9)
Injury, poisoning and procedural complications	Product packaging confusion	15	81.76 (49.15, 136.01)	81.46 (48.94, 135.6)	1183.13	6.34 (5.63)	80.85 (52.81)
Injury, poisoning and procedural complications	Maternal exposure during delivery	3	55.98 (17.99, 174.16)	55.94 (17.95, 174.35)	161.02	5.8 (4.38)	55.65 (21.53)
Cardiac disorders	Ventricular tachyarrhythmia	3	124.16 (39.76, 387.67)	124.07 (39.81, 386.7)	362	6.94 (5.51)	122.65 (47.3)
Cardiac disorders	Coronary artery thrombosis	15	112.21 (67.4, 186.79)	111.8 (67.16, 186.11)	1630.02	6.79 (6.08)	110.65 (72.23)
Cardiac disorders	Cardiac ventricular thrombosis	4	67.01 (25.06, 179.19)	66.95 (25.13, 178.39)	258.24	6.06 (4.79)	66.54 (29.22)
Cardiac disorders	Atrial thrombosis	9	55.83 (28.98, 107.56)	55.71 (29.18, 106.37)	481.05	5.79 (4.89)	55.43 (32.02)
Reproductive system and breast disorders	Ovarian vein thrombosis	3	309.79 (98.25, 976.78)	309.56 (97.39, 983.93)	896.58	8.23 (6.79)	300.83 (115.08)
Reproductive system and breast disorders	Perineal pain	5	116.42 (48.2, 281.21)	116.28 (48.13, 280.9)	565.28	6.85 (5.68)	115.04 (55)
Renal and urinary disorders	Ureteric perforation	5	1773.55 (687.78, 4573.35)	1771.38 (691.4, 4538.33)	7583.08	10.57 (9.32)	1518.47 (687.35)
Renal and urinary disorders	Renal cortical necrosis	8	575.62 (282.41, 1173.28)	574.5 (283.7, 1163.4)	4345.18	9.09 (8.12)	545.09 (300.4)
Investigations	Pco2 abnormal	4	1329.84 (470.08, 3762.01)	1328.54 (470.14, 3754.22)	4716.59	10.21 (8.86)	1181.03 (494.74)
Investigations	False negative investigation result	4	77.37 (28.92, 206.99)	77.3 (29.01, 205.96)	299.07	6.26 (4.99)	76.75 (33.69)
General disorders and administration site conditions	Vascular stent stenosis	5	184.74 (76.27, 447.47)	184.52 (76.38, 445.75)	897.06	7.5 (6.34)	181.39 (86.52)
General disorders and administration site conditions	Vascular stent thrombosis	14	89.51 (52.85, 151.6)	89.21 (52.55, 151.44)	1210.93	6.47 (5.73)	88.47 (56.93)
Hepatobiliary disorders	Hepatic cyst ruptured	3	1595.41 (473.9, 5371.1)	1594.25 (472.94, 5374.17)	4153.69	10.44 (8.91)	1386.43 (502.09)
Eye disorders	Retinal artery occlusion	16	96.34 (58.83, 157.75)	95.97 (58.79, 156.65)	1490.24	6.57 (5.88)	95.12 (62.96)

## 4 Discussion

TXA, among antifibrinolytic agents, has demonstrated efficacy in preventing bleeding complications across various hemostatic challenges while minimizing adverse effects (Hunt, 2015; Chornenki et al., 2019). The landmark WOMAN study illustrated the survival benefits of TXA in *postpartum* hemorrhage patients (WOMAN Trial Collaborators, 2017). Additionally, a systematic review revealed TXA’s ability to reduce blood loss in surgical patients by nearly one-third compared to placebo (Ker et al., 2013). The CRASH-2 trial further confirmed TXA’s effectiveness in acute traumatic hemorrhage, showing a one-third reduction in mortality when administered within 3 hours of the inciting event (Roberts et al., 2013).

In addition to these extensively researched indications, TXA has shown promising clinical benefits in various other areas. Some emerging fields currently under investigation include gastrointestinal bleeding, subdural and subarachnoid hemorrhage, spontaneous chronic urticaria, chemotherapy-induced thrombocytopenia, and ruptured abdominal aortic aneurysm (Cai et al., 2020). In clinical practice, TXA is widely applied across a spectrum of specialties, including obstetrics (WOMAN Trial Collaborators, 2017), acute trauma (Perel et al., 2013), orthopedic surgery (Elwatidy et al., 2008), cardiothoracic surgery (Myles et al., 2017), dental procedures (Myles et al., 2017), hemoptysis (Márquez-Martín et al., 2010), epistaxis (Zahed et al., 2018), as well as primary and secondary hemostatic disorders (Estcourt et al., 2016).

This study observed a significant increase in AERs related to TXA in recent years, with a notable surge in adverse reactions occurring in 2019. This trend aligns with the rising utilization rate and expanding indications of TXA during this period. Several studies suggest that 2019 may have been a period when the clinical application of TXA became more widely recognized (Ahmadzia et al., 2020; Sterling et al., 2023). TXA was more extensively used in 2019 to treat various types of bleeding symptoms, such as those resulting from surgeries, injuries, or *postpartum* haemorrhage (Bolliger and Tanaka, 2020; Bharath et al., 2019; CRASH-3 trial collaborators, 2019; Brenner et al., 2019). With the widening scope of medication usage, more patients received treatment, potentially accompanied by less cautious application by healthcare practitioners, thereby resulting in the notable increase in adverse reaction reports.

Additionally, AERs associated with TXA were more commonly reported among female patients compared to male patients. This could be attributed to females being more inclined to report AEs frequently or to the more prevalent use of TXA in treating conditions such as menorrhagia and *postpartum* hemorrhage among females. Regarding age, the distribution of reports across different age groups was relatively uniform, indicating no significant age bias in TXA adverse reactions. It is noteworthy that the majority of reports were submitted by healthcare professionals, ensuring the professionalism and authenticity of AERs. Since most reports originated from the United States, this may also reflect reporting trends specific to certain regions or cultures, necessitating further investigation to confirm potential regional or cultural biases. Finally, the majority of ADEs occurred within 7 days of medication use, with indications primarily associated with bleeding and menorrhagia, aligning with the pharmacological characteristics of TXA as an indirect fibrinolysis inhibitor and its primary indications.

AEs associated with TXA primarily involve Vascular disorders, Cardiac disorders, Nervous system disorders, Injury, poisoning, and procedural complications, as well as General disorders and administration site conditions. Of particular note, Nervous system disorders are specific to this medication and are not mentioned in the drug’s labeling, warranting further investigation and attention. At the PT level, higher rates of myoclonus, thrombosis, and epilepsy were observed. Additionally, this study identified AEs not documented in the drug’s labeling, such as ureter perforation, renal cortical necrosis, hepatic cyst rupture, and retinal artery occlusion. These AEs all exhibited high signals in quantitative signal detection, indicating a certain level of associated risk with the use of TXA.

TXA is available in both intravenous and oral formulations, acting as an antifibrinolytic agent by blocking lysine binding sites on plasminogen molecules. This action inhibits the interaction between plasminogen and formed plasmin and fibrin, thereby stabilizing the preformed fibrin meshwork generated during secondary hemostasis (McCormack, 2012; Stansfield et al., 2020; Wu et al., 2020). However, this mechanism also presents risks of thrombosis and subsequent vascular and cardiovascular complications, which may be inevitable. Nonetheless, studies suggest that early administration of TXA to trauma patients with significant bleeding or at risk of bleeding can reduce the risk of bleeding-related mortality without a notable increase in fatal or non-fatal thrombotic events. Additionally, tranexamic acid can significantly lower overall mortality (Shakur et al., 2010). Nevertheless, caution should be exercised when using TXA in patients with active intravascular coagulation, active thromboembolic disease, or an imbalance in the hemostatic system favoring thrombus formation, to prevent thrombus formation.

Compared to common AEs within the cardiovascular system, the strong association between TXA and AEs in the nervous system, particularly seizures, is unexpected and has not received widespread attention in drug labeling. There have been reports linking TXA to adverse neurological reactions, primarily occurring in the early postoperative period following cardiac surgery (de Leede-van der Maarl et al., 1999; Keyl et al., 2011; Koster et al., 2013). Several animal studies suggest that TXA’s proconvulsant properties may stem from its direct impact on the central nervous system. Administration of TXA to the cortex or injection into the brain ventricles of experimental animals can induce generalized seizures (Yamaura et al., 1980; Pellegrini et al., 1982; Schlag et al., 2002). Clinical research also supports this notion, indicating that TXA acts as a competitive antagonist of the receptors for glycine and gamma-aminobutyric acid (Furtmüller et al., 2002). Consequently, higher concentrations of TXA in the brain may heighten the risk of seizures. Studies further suggest that isoflurane or propofol could potentially prevent or treat early postoperative TXA-induced seizures (Mohseni et al., 2009; Kaabachi et al., 2011; Lecker et al., 2012), although additional research is warranted to mitigate the occurrence of severe neurological side effects.

Hemorrhagic rupture is a rare but life-threatening complication of simple hepatic cysts, often presenting with acute abdominal pain (Marion et al., 2013; Simon et al., 2015). Reports suggest that the acute administration of antithrombotic drugs in patients with hepatic cysts can exacerbate intraperitoneal hemorrhage and lead to hemodynamic instability (Tong et al., 2019). However, there are currently no other reported studies investigating the use of antifibrinolytic agents such as TXA in causing hepatic cyst rupture. For patients suspected of having hepatic cysts, abnormalities in liver function may accelerate intravascular coagulation and fibrinolysis (O'Leary et al., 2019). Therefore, during episodes of acute bleeding, extra caution should be exercised, with careful consideration given to the risk of hemorrhagic rupture.

TXA has been linked to ureter perforation and renal cortical necrosis, in line with relevant reports (Lee et al., 2022; Maresca et al., 2022). TXA treatment may induce the formation of urinary clots in hematuria patients, leading to secondary ureter perforation and other related adverse reactions, potentially culminating in renal failure (Lee et al., 2022). However, existing evidence has not conclusively demonstrated an increased risk of renal function failure in hematuria patients exposed to TXA. Further research is needed to explore this relationship.

Retinal artery occlusion is also a secondary adverse reaction caused by TXA-induced thrombosis, with limited relevant reports and the underlying mechanism remaining unclear (Parsons et al., 1988; Roumeliotis et al., 2020; Kiser et al., 2021). Healthcare professionals, both medical and non-medical, should be aware of this rare but potential AEs.

Finally, it’s important to note that many AEs result from errors in product administration, confusion in product appearance, and unclear product packaging. Therefore, there should be strengthened pharmaceutical regulation of TXA to prevent adverse reactions from occurring.

While this study offers robust scientific evidence for assessing the safety of TXA from various angles, it does have certain limitations. Primarily, the reliance on spontaneous reporting for data collection introduces potential biases and incomplete information. Consumer-reported data, for example, may not be as reliable or thorough as that provided by medical professionals. Moreover, there could be sampling biases in regions or countries with higher report numbers. Additionally, the data did not account for comorbidities associated with reported events, which could potentially confound any inference of causality between TXA and adverse events. Therefore, despite observing signals, establishing a causal relationship with TXA may prove challenging. Furthermore, limitations of the database, the retrospective nature of the study, and possible reporting biases could impact these findings. To achieve a more comprehensive and precise assessment, future research endeavors could explore employing stricter prospective methodologies, integrating clinical trials with epidemiological studies, thereby enhancing the accuracy of safety risk evaluations associated with TXA.

## 5 Conclusion

TXA stands as a cornerstone therapeutic agent in the management of hemorrhage across diverse medical specialties. While its efficacy in mitigating bleeding complications is well-established, comprehensive pharmacovigilance is imperative to discern and address potential adverse reactions associated with its administration. This study found that, apart from common adverse reactions, particular attention should be paid to adverse neurological effects, especially seizures. Additionally, caution is advised when administering TXA to patients with impaired liver or kidney function. Healthcare practitioners should be aware of the potential occurrence of rare thrombotic secondary adverse reactions. Lastly, there should be strengthened pharmaceutical regulation of TXA to prevent AEs.

## Data Availability

The original contributions presented in the study are included in the article/Supplementary Material, further inquiries can be directed to the corresponding authors.

## Abbreviations

ADEs, adverse drug events; AEs, adverse events; AERs, adverse event reports; ASCII, the American Standard Code for Information Interchange; BCPNN, Bayesian confidence propagation neural network; CRASH-2, Clinical Randomization of an Antifibrinolytic in Significant Hemorrhage; EBGM, empirical Bayes geometric mean; FAERS, FDA Adverse Event Reporting System; MedDRA, Medical Dictionary for Regulatory Activities; PRR, proportional reporting ratios; PS, primary suspect; PTs, preferred terms; ROR, reporting odds ratios; SOCs, System Organ Classes; TXA, Tranexamic acid.
